# Supplementary material for: Mobility Function and Aperiodic Electrocortical Activity in Younger and Older Adults
Source: IEEE Trans Neural Syst Rehabil Eng. Author manuscript; Available in PMC 2026 Jul 24. (PMC13398746; doi:10.1109/TNSRE.2026.3693634)
Supplement: supp1-3693634 [file NIHMS2179444-supplement-supp1-3693634.pdf]

Mobility Function and Aperiodic Electrocortical Activity in Younger and Older Adults

Supplemental Materials

Charlotte R. DeVol, Chang Liu, *Member, IEEE*, Jacob Salminen, Erika M. Pliner, Arkaprava Roy, Chris J. Hass, David J. Clark, Todd M. Manini, Rachael D. Seidler, Daniel P. Ferris, *Senior Member, IEEE*

Supplemental Table I. Data retention after key processing steps for younger and older adults. Values reported as mean ± standard deviation

	Younger Adults	Older Adults	p-value
Number of channels retained for each participant after *clean_artifacts*	108 ± 6	111 ± 6	0.07
Percent of total time removed with *clean_artifacts**	1.00 ± 1.29	1.39 ± 2.06	0.77
Number of brain components retained for each participant	15 ± 5	12 ± 5	0.01

*Percent of total time removed was restricted to never exceed 10%, but this maximum was not reached in any participants.

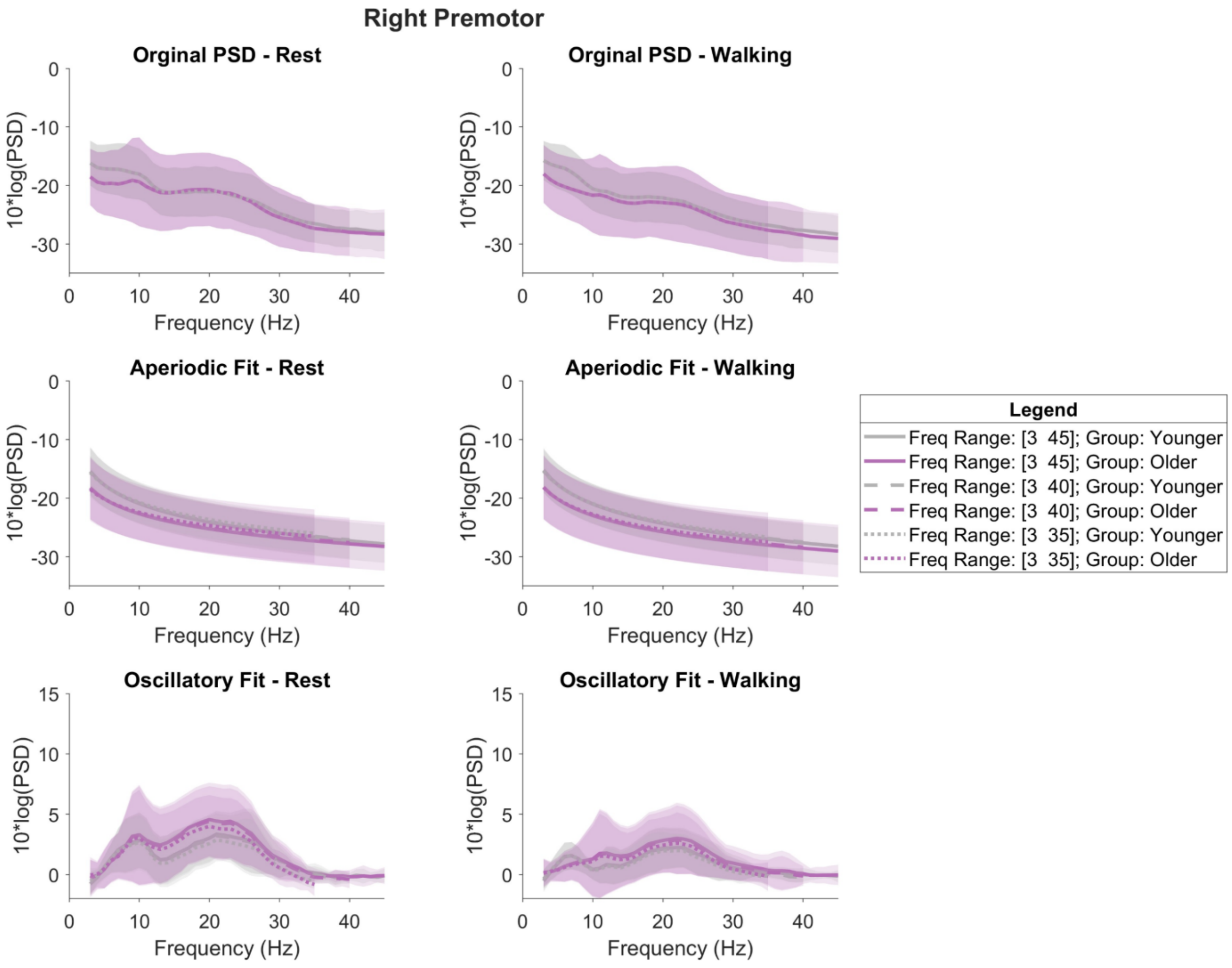

Supplemental Fig. 1. The right premotor region's original power spectral density plots (PSDs), aperiodic fit, and oscillatory fit during rest and walking for three different frequency ranges: 3-35 Hz (small dashed line), 3-40 Hz (large dashed line), and 3-45 Hz (solid line). Grey is the younger adults, and purple is the older adults. Plots indicate the group mean and standard deviation. Axis limits are held consistent across all figures showing the same results for different brain regions (Supplemental Figures 1-8).

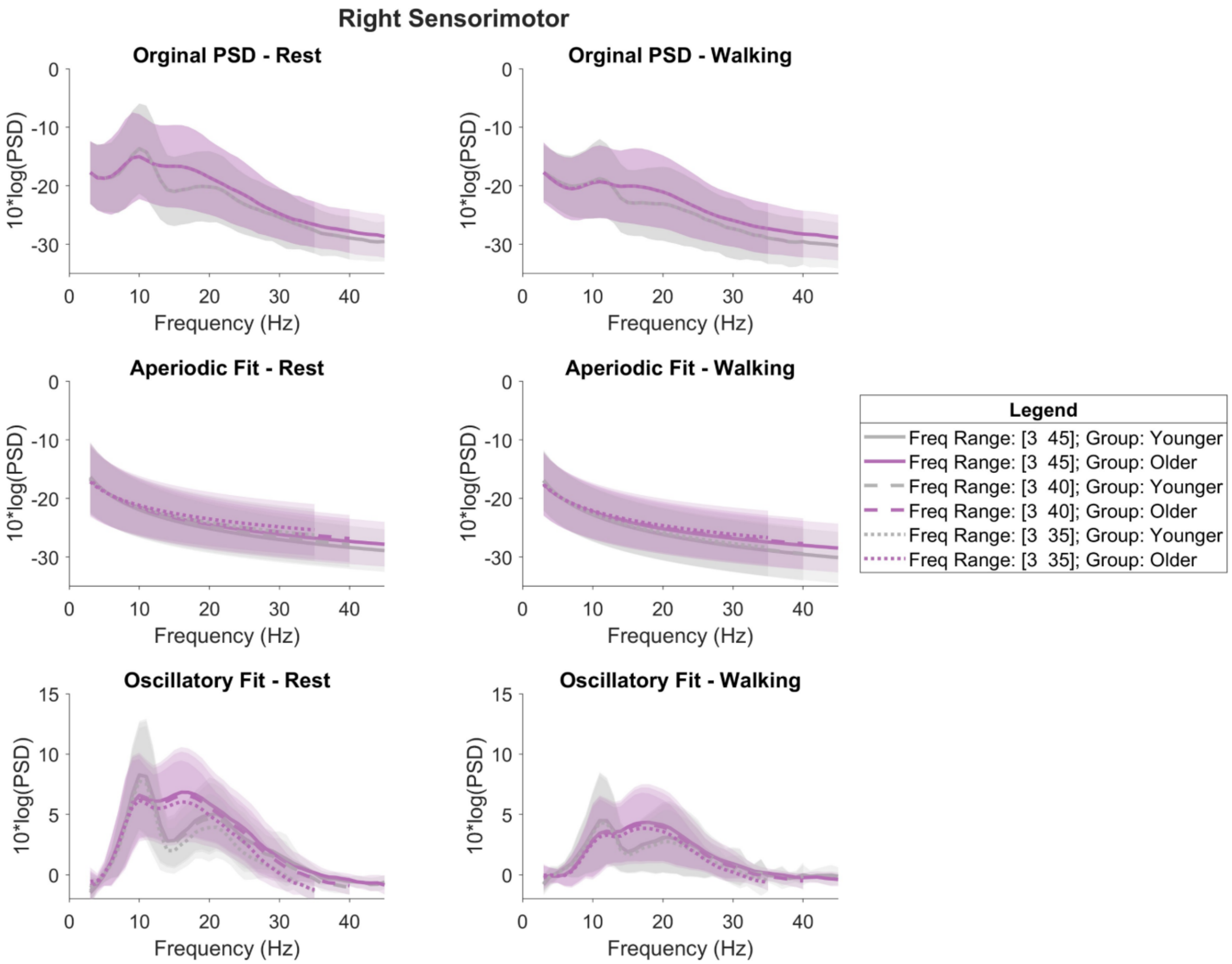

Supplemental Fig. 2. The right sensorimotor region's original power spectral density plots (PSDs), aperiodic fit, and oscillatory fit during rest and walking for three different frequency ranges: 3-35 Hz (small dashed line), 3-40 Hz (large dashed line), and 3-45 Hz (solid line). Grey is the younger adults, and purple is the older adults. Plots indicate the group mean and standard deviation. Axis limits are held consistent across all figures showing the same results for different brain regions (Supplemental Figures 1-8).

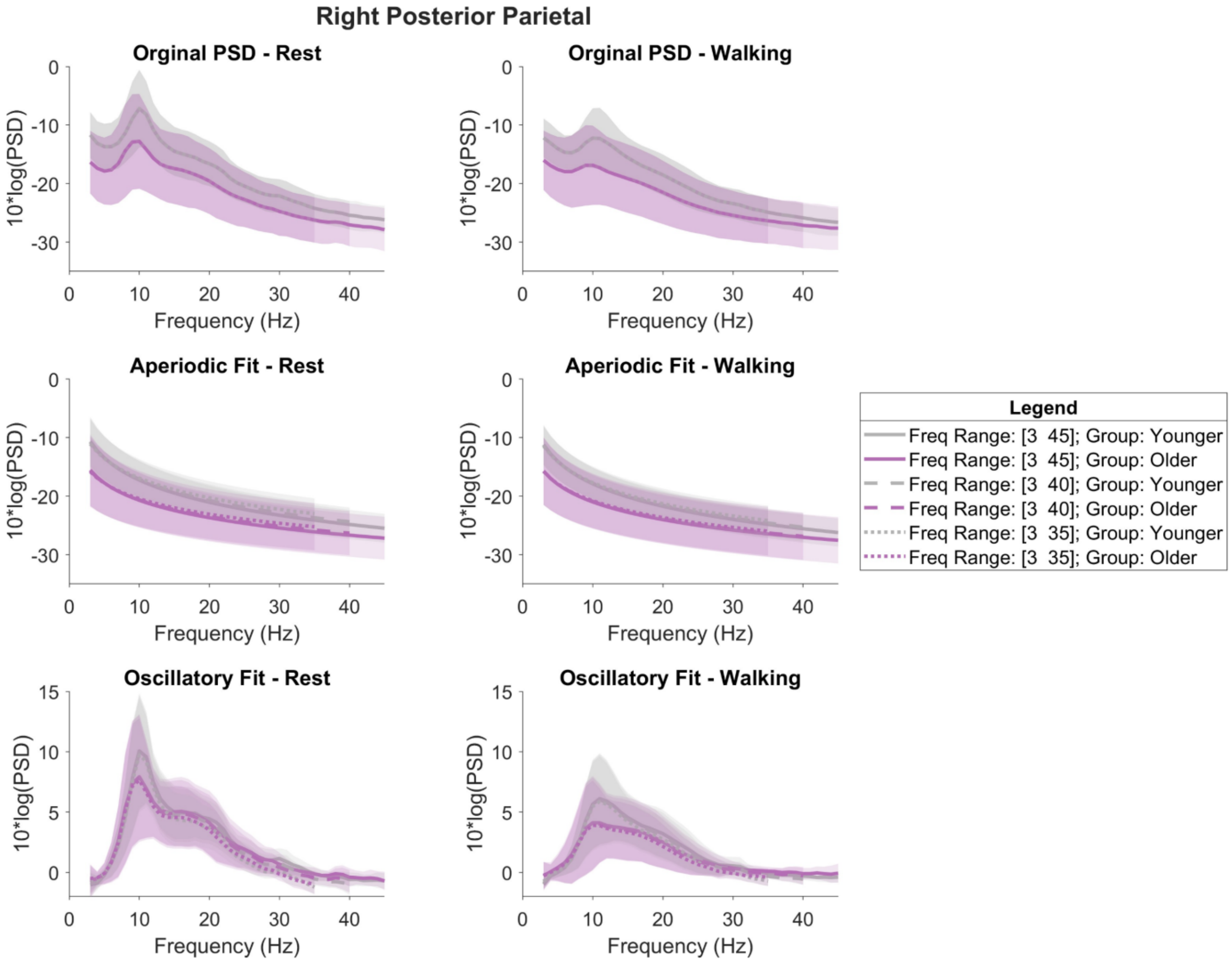

Supplemental Fig. 3. The right posterior parietal region's original power spectral density plots (PSDs), aperiodic fit, and oscillatory fit during rest and walking for three different frequency ranges: 3-35 Hz (small dashed line), 3-40 Hz (large dashed line), and 3-45 Hz (solid line). Grey is the younger adults, and purple is the older adults. Plots indicate the group mean and standard deviation. Axis limits are held consistent across all figures showing the same results for different brain regions (Supplemental Figures 1-8).

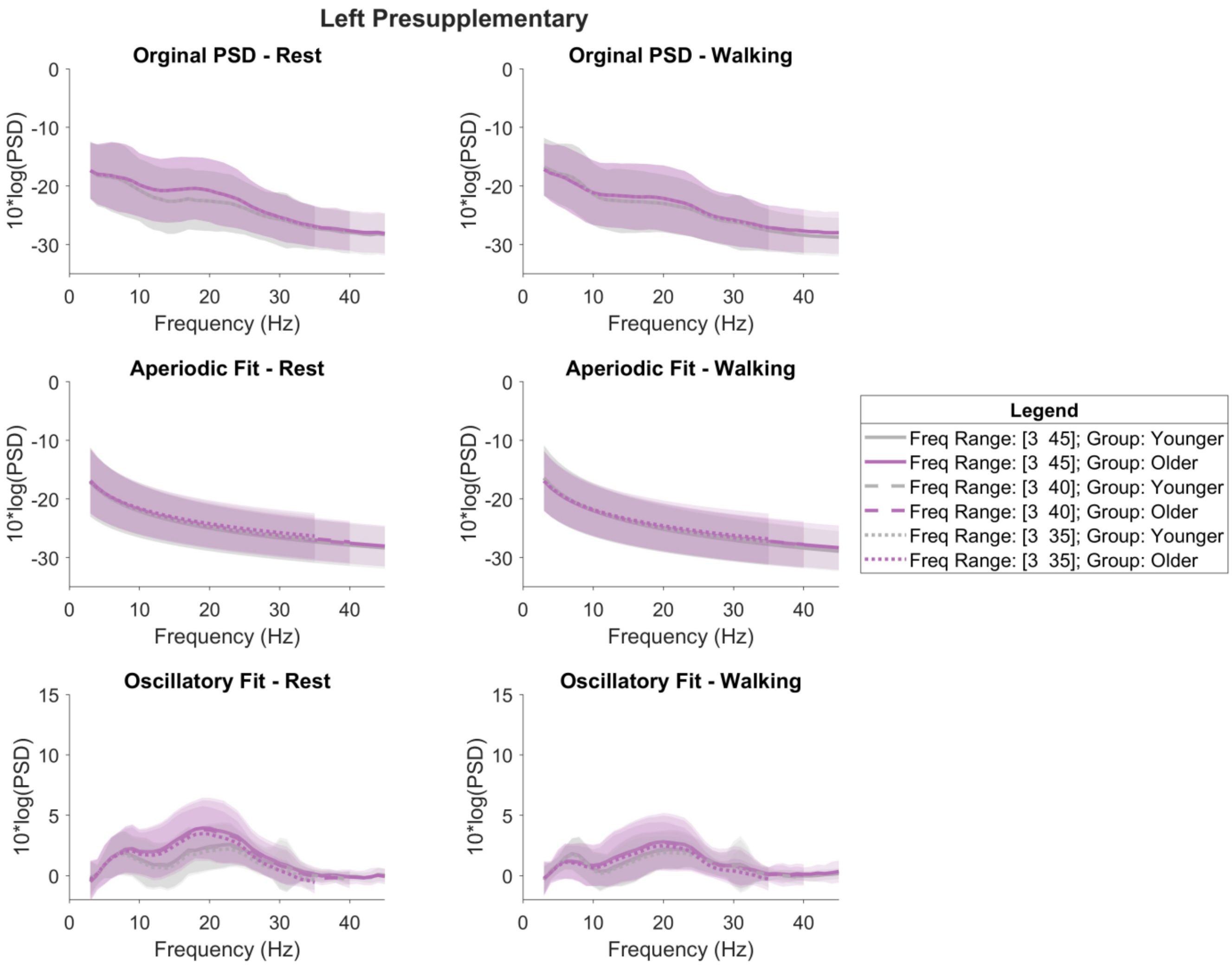

Supplemental Fig. 4. The left presupplementary region's original power spectral density plots (PSDs), aperiodic fit, and oscillatory fit during rest and walking for three different frequency ranges: 3-35 Hz (small dashed line), 3-40 Hz (large dashed line), and 3-45 Hz (solid line). Grey is the younger adults, and purple is the older adults. Plots indicate the group mean and standard deviation. Axis limits are held consistent across all figures showing the same results for different brain regions (Supplemental Figures 1-8).

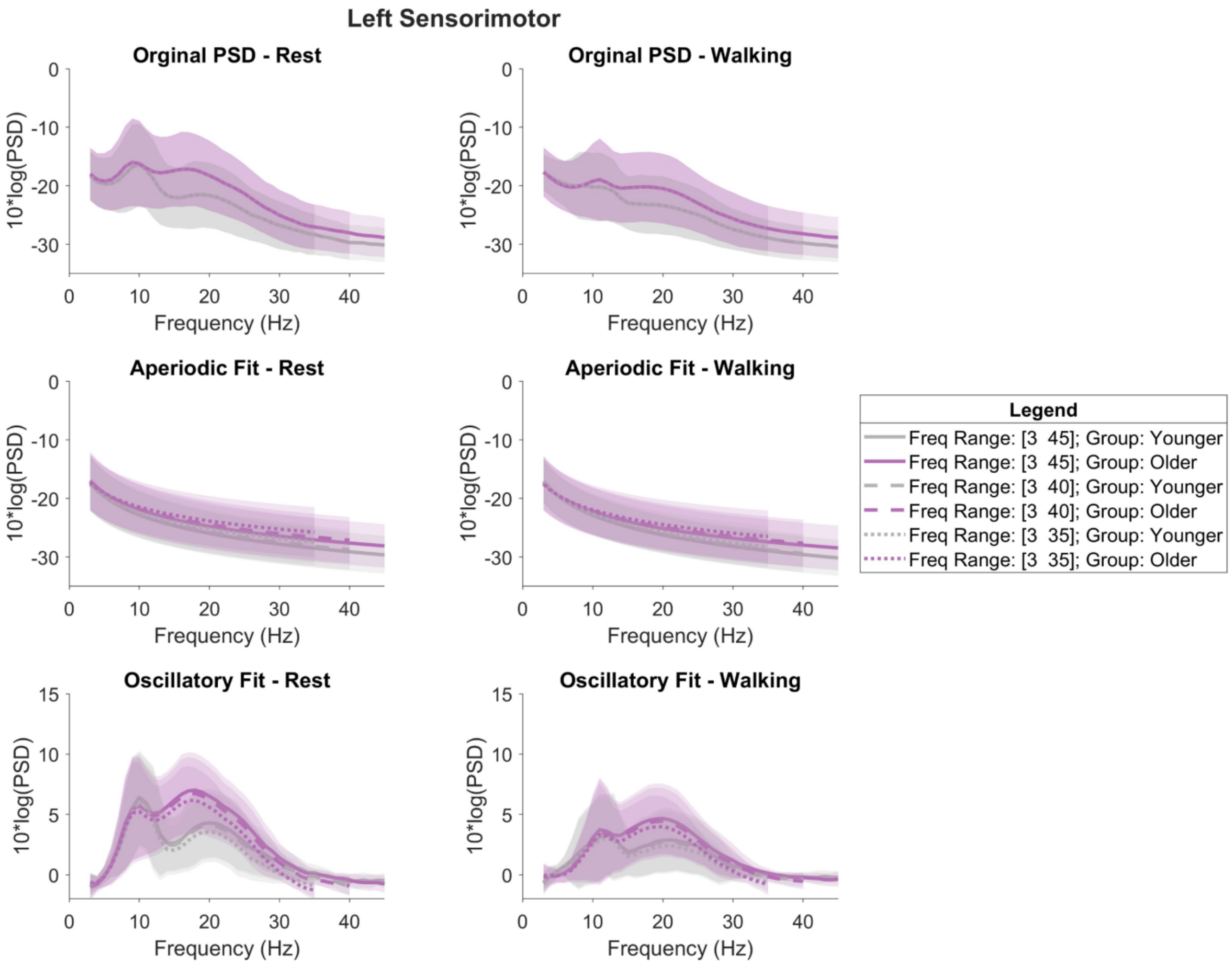

Supplemental Fig. 5. The left sensorimotor region's original power spectral density plots (PSDs), aperiodic fit, and oscillatory fit during rest and walking for three different frequency ranges: 3-35 Hz (small dashed line), 3-40 Hz (large dashed line), and 3-45 Hz (solid line). Grey is the younger adults, and purple is the older adults. Plots indicate the group mean and standard deviation. Axis limits are held consistent across all figures showing the same results for different brain regions (Supplemental Figures 1-8).

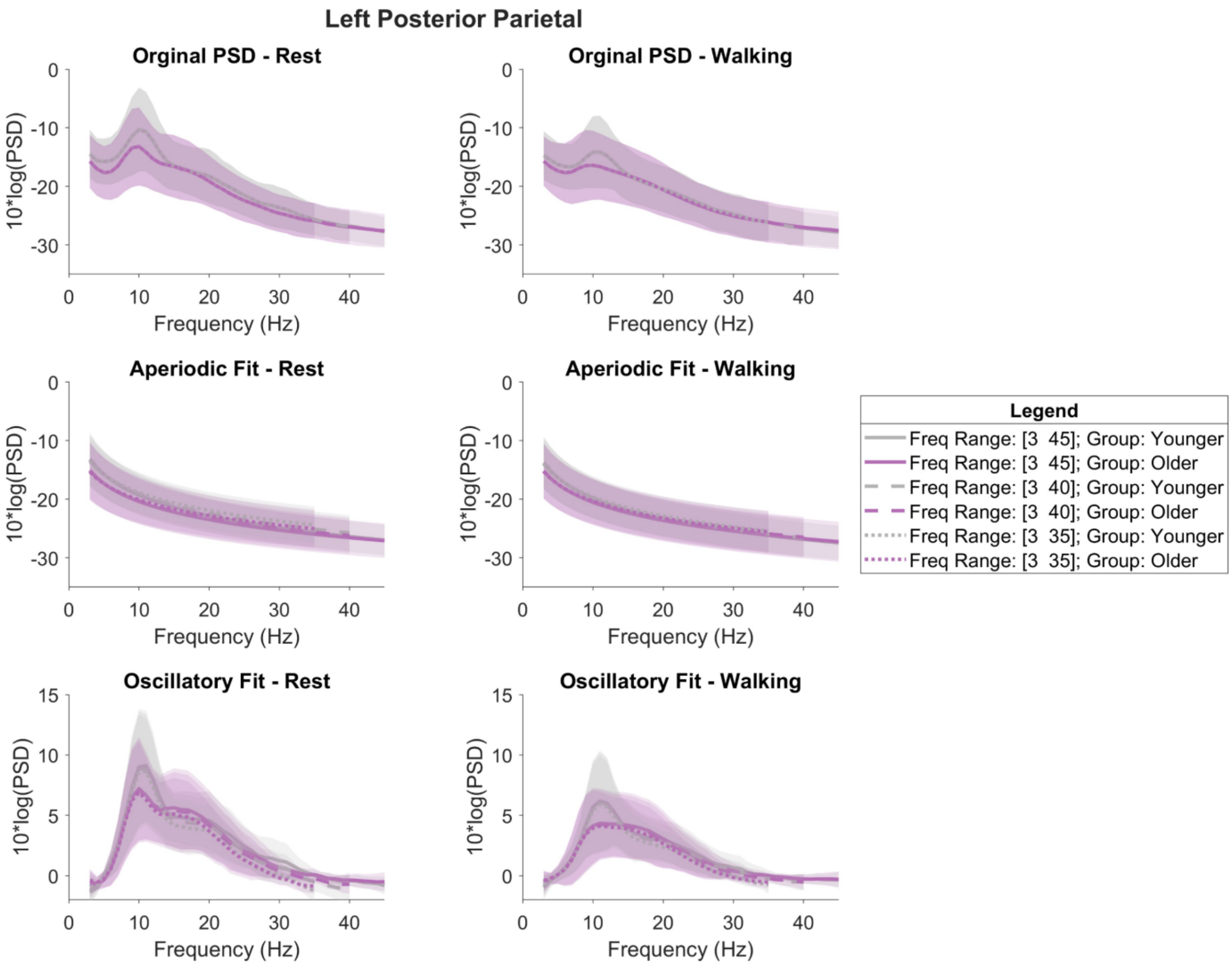

Supplemental Fig. 6. The left posterior parietal region's original power spectral density plots (PSDs), aperiodic fit, and oscillatory fit during rest and walking for three different frequency ranges: 3-35 Hz (small dashed line), 3-40 Hz (large dashed line), and 3-45 Hz (solid line). Grey is the younger adults, and purple is the older adults. Plots indicate the group mean and standard deviation. Axis limits are held consistent across all figures showing the same results for different brain regions (Supplemental Figures 1-8).

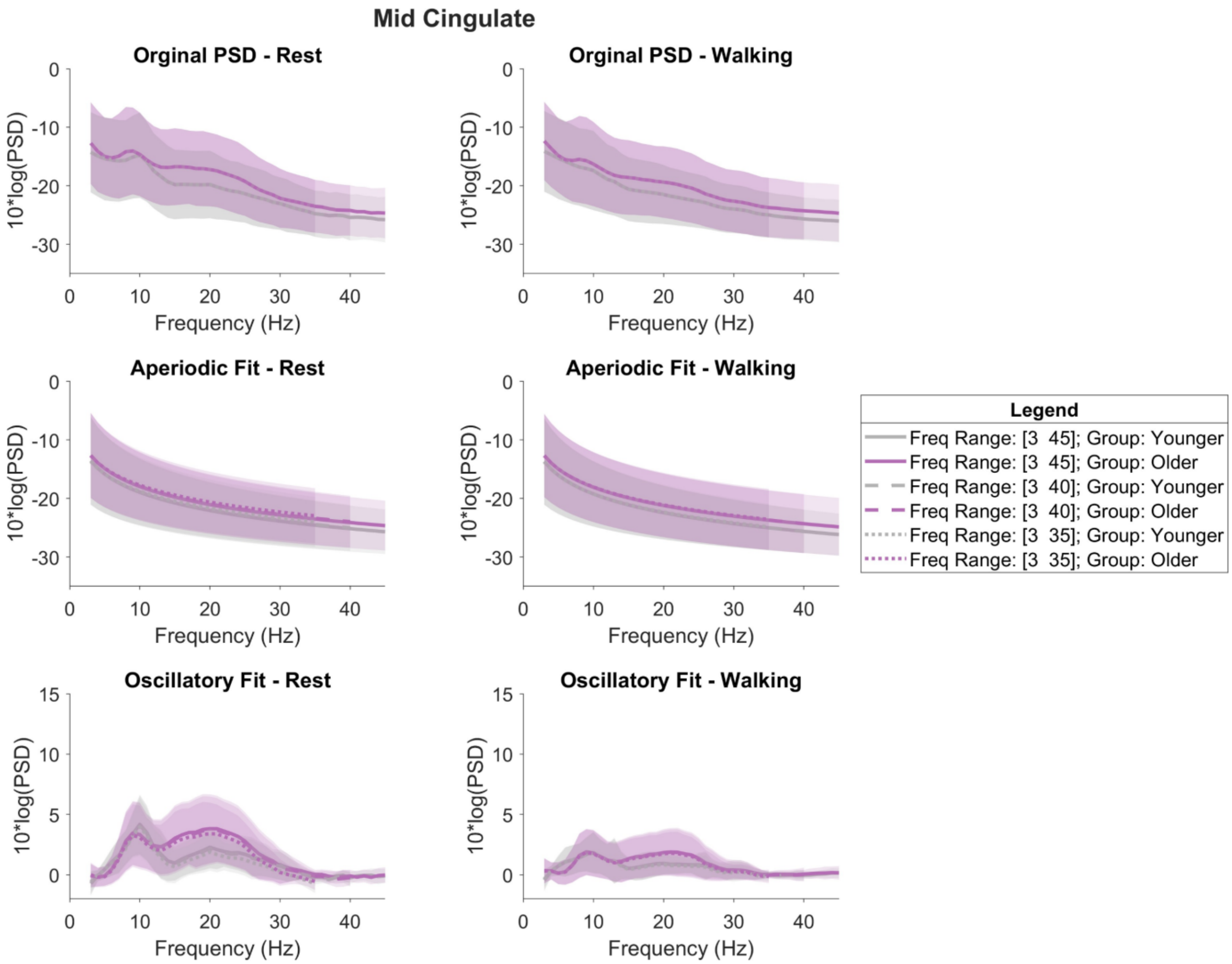

Supplemental Fig. 7. The mid cingulate region's original power spectral density plots (PSDs), aperiodic fit, and oscillatory fit during rest and walking for three different frequency ranges: 3-35 Hz (small dashed line), 3-40 Hz (large dashed line), and 3-45 Hz (solid line). Grey is the younger adults, and purple is the older adults. Plots indicate the group mean and standard deviation. Axis limits are held consistent across all figures showing the same results for different brain regions (Supplemental Figures 1-8).

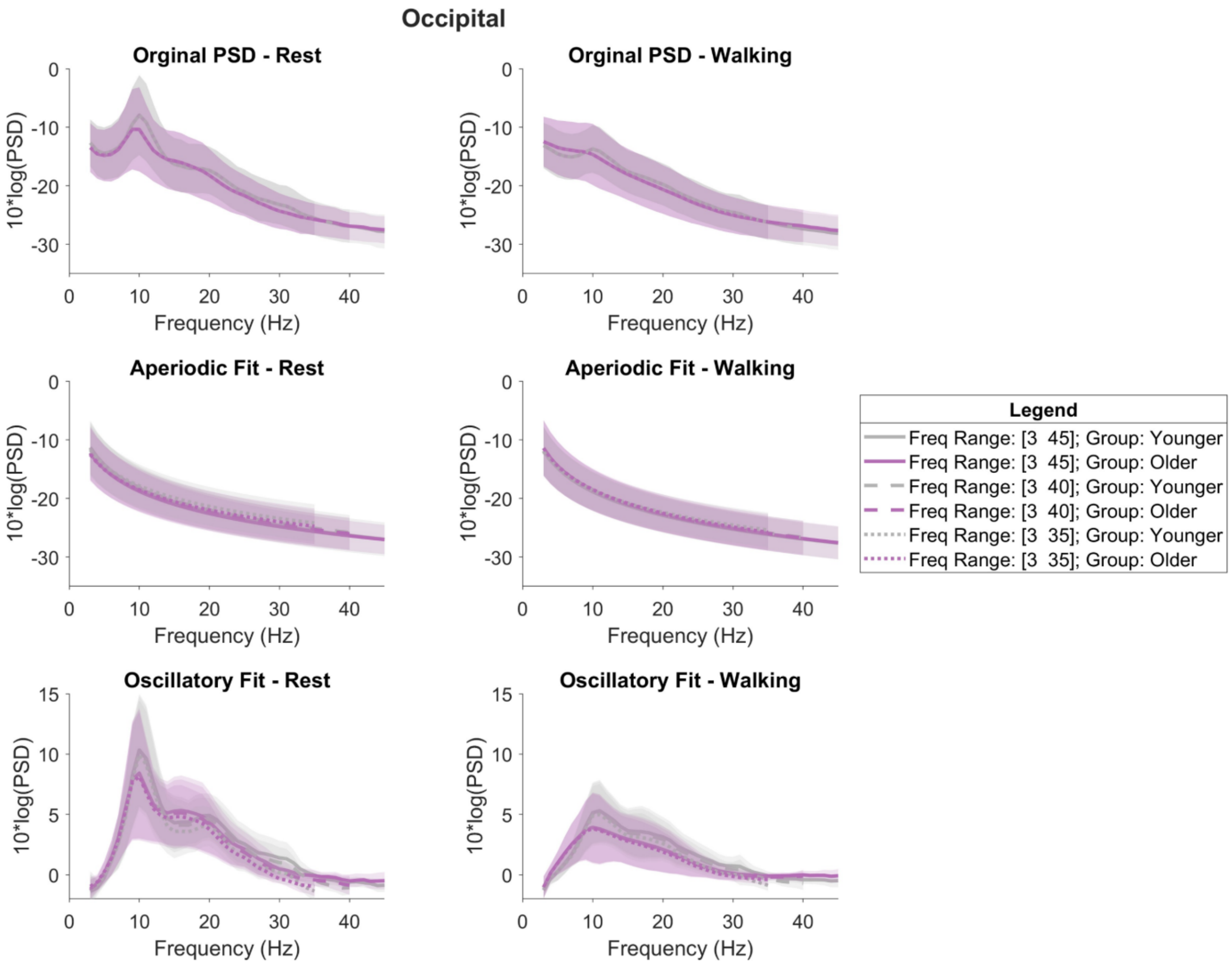

Supplemental Fig. 8. The occipital region's original power spectral density plots (PSDs), aperiodic fit, and oscillatory fit during rest and walking for three different frequency ranges: 3-35 Hz (small dashed line), 3-40 Hz (large dashed line), and 3-45 Hz (solid line). Grey is the younger adults, and purple is the older adults. Plots indicate the group mean and standard deviation. Axis limits are held consistent across all figures showing the same results for different brain regions (Supplemental Figures 1-8).

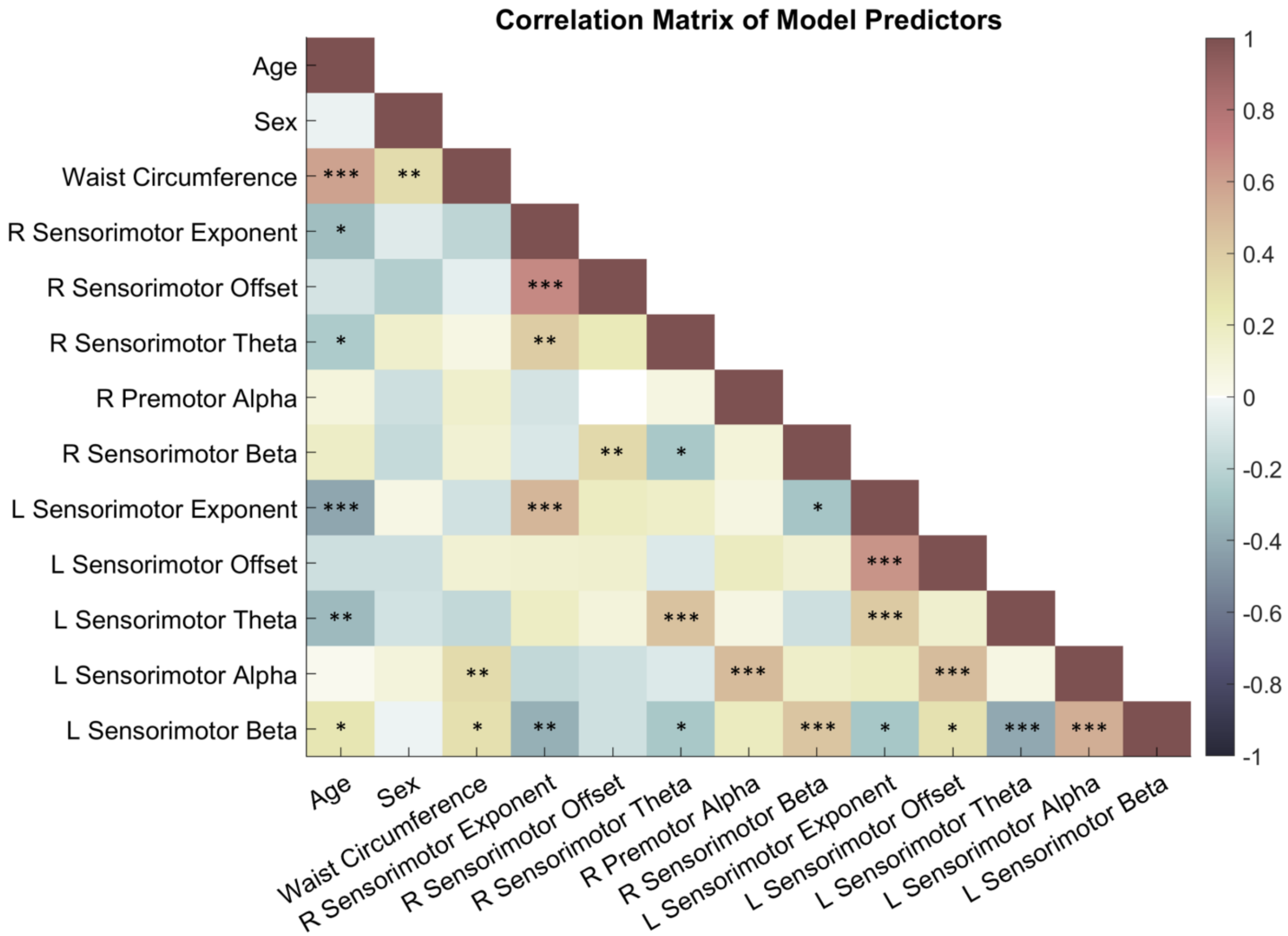

Supplemental Fig. 9. Correlogram of model predictors used to generate the Bayesian Additive Regression Tree (BART) model. The correlogram is given to aid in interpreting ALE plots. ALE plots only represent the relationship between each BART model predictor and the response variable (individualized walking speeds) given the relationships that exist in the data set. The strongest correlations were seen between the aperiodic offset and exponent for each brain region. Asterisks indicate the p-value from each correlation: *** $p < 0.001$; ** $p < 0.01$; * $p < 0.05$.
